# Unveiling the Silent Tear: A 35-Year-Old With Isolated Spontaneous Celiac Artery Dissection

**DOI:** 10.7759/cureus.105462

**Published:** 2026-03-18

**Authors:** Affan Ahmed, Ravi Patel, Colin Ly

**Affiliations:** 1 Internal Medicine, Methodist Dallas Medical Center, Dallas, USA

**Keywords:** arterial dissection, bowel ischemia, celiac artery dissection, endovascular intervention, epigastric pain, hyperlipidemia, hypertension, vascular disorders, vascular surgery, vasculitis

## Abstract

Isolated spontaneous celiac artery dissection is a rare condition with only a few reported cases, especially in individuals with minimal risk factors. Celiac artery dissection has been associated with several risk factors, including hypertension, vasculitis, pregnancy, connective tissue disease, smoking, diabetes, and dyslipidemia. The condition typically affects middle-aged men, with a mean age of 55 years. We present a case of celiac artery dissection in a young 35-year-old man with a past medical history of obesity and mild dyslipidemia. The patient presented with spontaneous mid-epigastric pain radiating to his left upper abdomen. This case of an isolated celiac artery dissection in a young patient without significant risk factors highlights the importance of maintaining a high clinical suspicion for this rare condition, even in otherwise healthy individuals presenting with acute abdominal pain.

## Introduction

Isolated spontaneous celiac artery dissection can be an unusual presentation for abdominal pain, especially in those with minimal risk factors. Celiac artery dissections are a rare differential for acute abdominal pain. It can also sometimes be found as an incidental finding. The celiac artery is one of the many vessels that branch off the abdominal aorta. It gives rise to other abdominal vessels that help supply organs such as the abdominal esophagus, liver, stomach, spleen, gallbladder, duodenum, and pancreas. It has three major branches, which are the left gastric, splenic, and common hepatic artery. The anatomy of the celiac trunk does have clinical relevance [1]. The reason why it is important to correctly recognize celiac artery dissections is because of their valuable role in supplying blood flow to essential organs. The etiology of celiac artery dissections can be multifactorial; some of these factors include inflammation, atherosclerotic disease, congenital disorders, trauma, or iatrogenic [2]. Isolated celiac artery dissections are rare, with some studies even estimating their incidence to be less than 0.1% in autopsy studies [3]. There are quite a few risk factors for celiac artery dissection. Studies have found that the most contributing risk factors are hypertension and smoking. Other risk factors that may also play a role include diabetes and dyslipidemia [4]. The pathophysiology of the disease is thought to arise from an intimal tear in the celiac trunk, which allows blood to split the arterial wall. This creates a false lumen, which then compresses the true lumen and impairs blood flow to foregut organs [5]. It is essential to recognize the signs and symptoms of celiac artery dissections as they can have serious complications such as visceral organ ischemia or infarction from compromised blood flow, aneurysmal dilation/pseudoaneurysm formation with risk of rupture and hemorrhage, and propagation of the dissection into branch vessels such as the splenic or hepatic arteries [6].

## Case presentation

**Figure 1:**
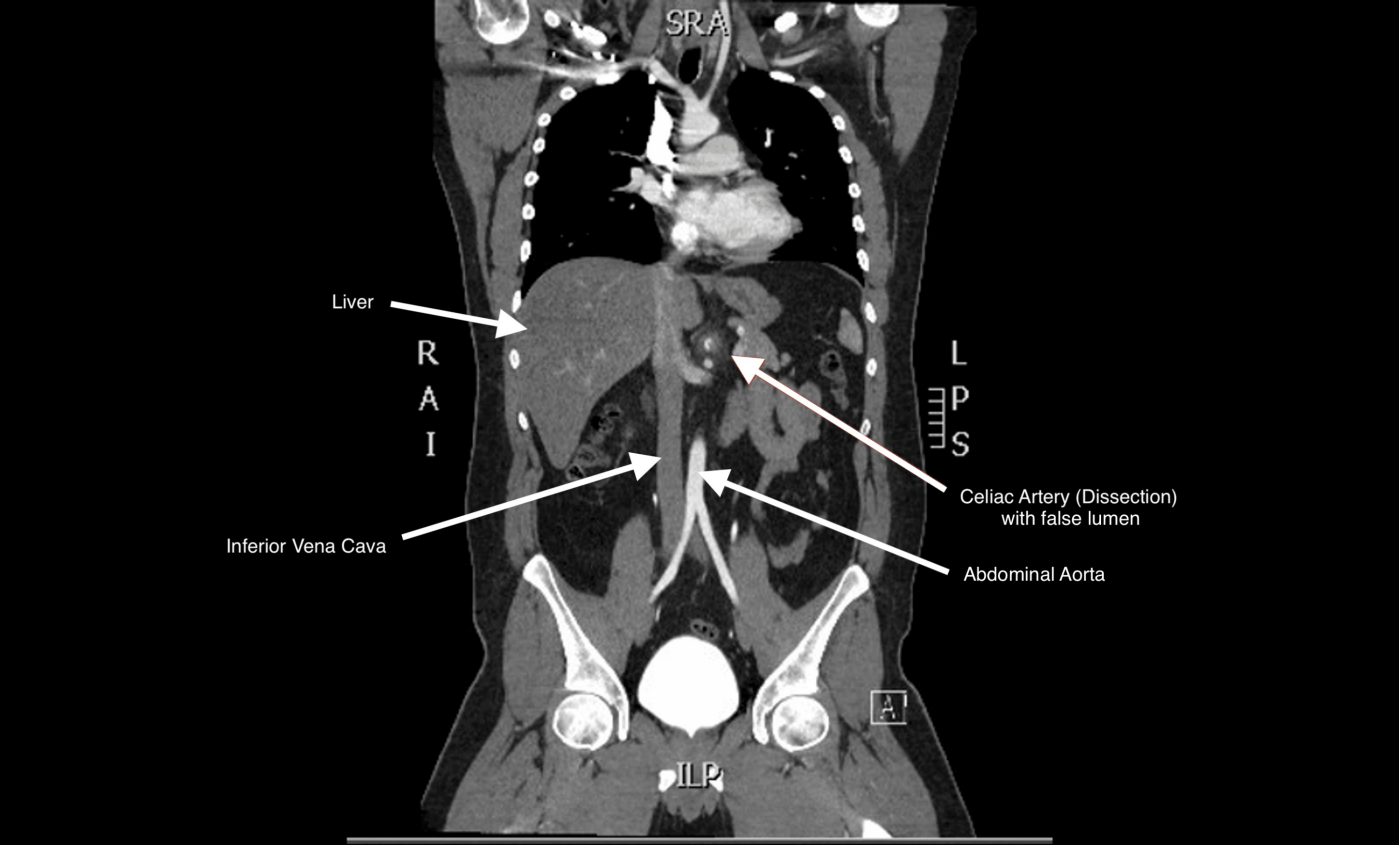
Computed tomography scan in coronal view

**Figure 2:**
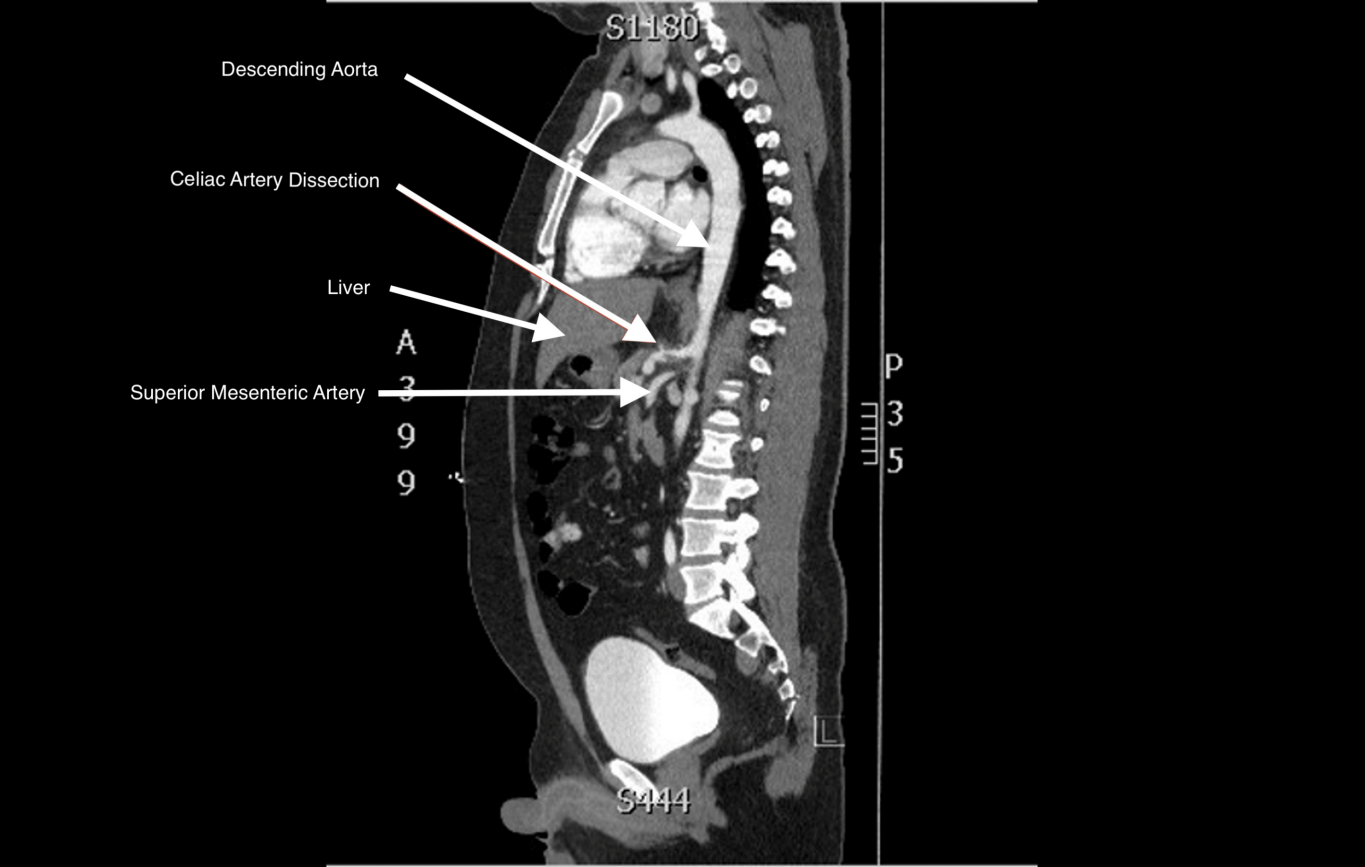
Computed tomography scan in the candy cane view

We present a case of celiac artery dissection in a young 35-year-old man with a past medical history of dyslipidemia (low-density lipoprotein (LDL) 112) and obesity (BMI 33.25) who developed spontaneous abdominal pain three days prior to his emergency department visit. The patient came in with concerns of sharp mid-epigastric abdominal pain with radiation to his left side. The patient denied any fever or vomiting but did admit to nausea. At its worst, the pain was a 10/10. On admission, the patient's vital signs were heart rate (HR) 72 beats per minute, blood pressure (BP) 150/94 mmHg, temperature (T) 98.1°F, respiratory rate (RR) 15 breaths per minute, and oxygen saturation (O_2_) 95%. On physical examination, the patient had generalized abdominal tenderness, with most of the pain localized to the epigastric region. Labwork on admission was unrevealing. He had a negative troponin, N-terminal pro-B-type natriuretic peptide (NT-proBNP), and erythrocyte sedimentation rate (ESR). His lactic acid was negative at 0.8, and he had a normal white cell count at 6,700. The rest of his labwork on admission was unremarkable as well. Computed tomography angiogram (CTA) of the aorta was ordered, which revealed: “inflammatory changes around the celiac artery with wall thickening and thrombus that results in moderate stenosis of the celiac artery.” Figure 1 shows the CTA of the dissection in the coronal view. Figure 2 shows the same finding in the “candy cane” view of the aorta. Vascular surgery later concluded that there was also an underlying isolated celiac artery dissection upon independent review of imaging. The hypercoagulability and vasculitis workups were unrevealing. The patient was started on a heparin drip, and a transthoracic echocardiogram revealed no underlying pathology. The patient had a repeat CTA 48 hours after, which revealed a subtle linear structure just distal to the thrombus, which suggested dissection with a thrombus secondary to a thrombosed false lumen in the celiac artery. He was discharged with oral anticoagulation and instructed to follow up with the vascular surgery outpatient.

## Discussion

A 35-year-old man presented with acute abdominal pain, and imaging findings were consistent with a celiac artery dissection. It is essential to recognize celiac artery dissections in patients who present with acute abdominal pain, especially isolated spontaneous celiac artery dissection, as seen in this patient. Isolated spontaneous celiac artery dissection is a rare entity that was first described in 1947 [7]. It typically affects males more often than females, with 80% of cases reported in men in a literature review conducted between 2004 and 2015. The mean age at diagnosis was 56.4 ± 10.4 years (range, 42-77 years) [8]. The present case is unique because it involves an isolated celiac artery dissection in a young 35-year-old man without significant contributing environmental factors or past medical history, aside from secondary dyslipidemia with an LDL level of 112 mg/dL. He had no history of hypertension or smoking, both of which are known major risk factors for celiac artery dissection. He was also not exposed to other environmental factors associated with this condition, such as tobacco use [9]. On admission, his vital signs were stable, with no concern for hemodynamic instability. Laboratory results were unremarkable. The workup for spontaneous isolated celiac artery dissection typically includes evaluation for hypercoagulable states, lipid profile, hemoglobin A1c, and abdominal imaging. Initial imaging noted only the thrombus; however, an independent review by the surgical team subsequently identified a celiac artery dissection. Abdominal imaging (Figures 1, 2) revealed dilation of the celiac artery, which is commonly seen due to altered hemodynamics distal to the obstruction. Figure 1 also demonstrates the false lumen of the dissection, creating the characteristic crescent-shaped appearance. Classification of celiac artery dissection can be performed using the Sakamoto classification system, which categorizes dissections based on the characteristics of the false lumen. This system divides dissections into four types depending on whether the false lumen is patent, thrombosed, or associated with aneurysmal dilation, helping guide prognosis and management decisions. This patient’s presentation appears to correspond to Class III in the Sakamoto classification [10]. It is also essential to rule out end-organ ischemia in the setting of celiac artery dissection; this can be assessed by measuring lactic acid levels. In this case, the patient’s lactic acid level remained within normal limits. Management of celiac artery dissection is typically conservative and may include antiplatelet therapy, anticoagulation, or both, although some cases require surgical intervention [11]. Anticoagulation was favored in this case because of the presence of an intraluminal thrombus and a suspected thrombosed false lumen. Anticoagulation is commonly used in spontaneous visceral artery dissections to prevent thrombus propagation and distal embolization while preserving true lumen patency. Prior studies suggest that anticoagulation is often selected in cases with thrombus or luminal narrowing, whereas antiplatelet therapy may be reserved for uncomplicated dissections [12]. Management decisions are made on a case-by-case basis, and evidence of end-organ ischemia may favor surgical intervention over medical management. Previous studies suggest that the most effective treatment strategies include preventing expansion of the dissecting hematoma, anticoagulation to prevent complications, and strict blood pressure control [2]. One limitation of this report is the lack of long-term follow-up after discharge, which prevents evaluation of the progression of the dissection and the patient’s response to medical management.

## Conclusions

When patients present with abdominal pain and a dissection is suspected, an aortic dissection is typically considered initially, while variations of arterial dissection, including those involving the celiac artery, are often overlooked. This case of an isolated celiac artery dissection in a young patient without significant risk factors highlights the importance of maintaining a high clinical suspicion for this rare condition, even in otherwise healthy individuals presenting with acute abdominal pain. One major limitation of this case report was the long-term effectiveness of the provided therapy. The patient was discharged after being stabilized and told to follow up with vascular surgery as an outpatient. Due to the lack of access to the patient’s outpatient records, the study was limited in its ability to assess the long-term outcomes of celiac artery dissections. The patient's only noted risk factor was secondary dyslipidemia, in the absence of common contributors such as hypertension, vasculitis, trauma, connective tissue disorders, or vascular disease. This unique presentation underscores the need for a thorough diagnostic workup to ensure prompt recognition and appropriate management of celiac artery dissection, which can have serious complications such as visceral organ ischemia, aneurysmal dilation/pseudoaneurysm formation with risk of rupture, and hemorrhage if not addressed promptly.
